# Transfusion Complications in Thalassemia: Patient Knowledge and Perspectives

**DOI:** 10.3389/fmed.2022.772886

**Published:** 2022-03-01

**Authors:** Sashoy Patterson, Ashley Singleton, Jane Branscomb, Vivien Nsonwu, Regena Spratling

**Affiliations:** ^1^Georgia Health Policy Center, Andrew Young School of Policy Studies, Georgia State University, Atlanta, GA, United States; ^2^School of Medicine, Meharry Medical College, Nashville, TN, United States; ^3^Byrdine F. Lewis College of Nursing and Health Professions, Georgia State University, Atlanta, GA, United States

**Keywords:** thalassemia, transfusion, transfusion guidelines, transfusion reactions, transfusion complications, patient knowledge

## Abstract

Chronic transfusion, used to manage clinically significant forms of thalassemia, carries complication risks including iron overload, alloimmunization, and allergic or hemolytic reactions. Dissemination and implementation of evidence-based guidelines for minimizing these risks are complicated by the small numbers and increasing dispersion of the thalassemia population. This elevates patients' role in understanding and communicating with providers about treatment. The present study sought to assess thalassemia patients' knowledge of transfusion, complications, and guidelines; their experience of clinicians' following guidelines; and their perspectives on ways to store and share personal transfusion data. A convenience sample of 32 patients participated in structured interviews. Most, if not all, understood iron overload, chelation therapy, blood typing, and cross-matching. Awareness of each of five of evidence-based transfusion guidelines that were discussed ranged from 72 to 88%. Patients expressed privacy concerns about each of three data storage options, slightly less for a registry than for a wallet card or smartphone app. The registry also avoided concerns that the other options placed extra burden on patients. Recommendations include increased education on the meaning and significance of packed, washed red blood cells, leuko-reduction, and detailed cross-matching, and implementation of a nationwide registry to make transfusion data available to providers anywhere at the point of care. Registry implementation should be sensitive to patients' privacy and security concerns, but also help them appropriately weigh those against safety benefits. These actions could help reduce transfusion complications in thalassemia by improving patient efficacy and increasing adherence to evidence-based guidelines.

## Introduction

Thalassemia describes a group of inherited blood disorders associated with the alpha and beta globulins of hemoglobin. Alpha thalassemia and beta thalassemia result from the reduction of either alpha or beta globulins, respectively. Both types of thalassemia result in moderate to severe anemia and extramedullary hematopoiesis, leading to signs and symptoms such as poor growth and development; skeletal deformities; thrombosis; pain in the head, back, and legs; impaired cardiac function; hepatosplenomegaly; non-transfusional iron overload; and conditions such as hydrops fetalis (1–4). It is estimated that 300,000 to 400,000 babies are born worldwide with hemoglobinopathies, with approximately 23,000 per year characterized as thalassemia major, the most severe form of thalassemia (5). Though cases of thalassemia worldwide typically concentrate around the “thalassemia belt,” immigration into the United States from Italy, Greece, Pakistan, Iran, India, and particularly central and southeast Asian countries has markedly increased the prevalence of thalassemia in this country (6–8).

Alpha thalassemia major is often incompatible with life. In cases of severe anemia in beta thalassemia major, individuals are usually diagnosed within the first 2 years of life and may survive with chronic, life-long blood transfusions (1, 3, 5, 9, 10). Unfortunately, transfusions have risks and complications. Iron overload and resulting endocrine complications, erythrocyte alloimmunization, transfusion reactions, and infections are all risks for thalassemia patients who are dependent on blood transfusions (5, 8). These complications, however, are largely preventable (11). If patients know their personal transfusion histories and are aware of key clinical practice recommendations, they can contribute to reducing their complication risk through their actions and through their communications with providers (3, 4, 8).

Little literature exists on thalassemia patients' self-reported knowledge and experience regarding transfusion complications and practices to lower risk. One study described the physical, psychosocial, and economic impacts of transfusion reported by 32 thalassemia patients, as well as challenges those patients encountered during transfusion procedures (12). The authors did not identify any studies addressing patient knowledge specific to transfusion complications, related practice guidelines, or patients' preference of data storage methods to improve transfusion safety.

The current study addresses these gaps in evidence, seeking to (1) describe knowledge and experience of transfusion among transfused thalassemia patients to inform patient education and outreach; (2) characterize patients' transfusion experience in relation to providers' adherence to recommended practices to inform provider education and outreach; and (3) assess patients' preferences regarding personal data tracking to inform methods for ensuring that providers have access to current, accurate transfusion histories to inform real-time decisions. The study's aim is to improve dissemination and implementation of best practices for reducing the risk of transfusion complications in thalassemia.

## Methods

### Study Design

This descriptive study collected data from individuals with thalassemia in the United States who received prior therapeutic transfusions. Structured interviews included questions eliciting participants' knowledge and experience of transfusions; their experience of providers' performance of key actions recommended in applicable guidelines published by the Thalassemia International Federation [TIF; (13)]; and their preferences and feedback regarding possible ways to store and share their personal transfusion data.

The interview guide (see Supplementary Material) was developed by an interdisciplinary team including thalassemia and transfusion medicine clinical specialists, qualitative researchers, and thalassemia patient advocates. It consisted of three parts: (1) six open-ended questions about patient knowledge and experience of transfusions; (2) patient review and reflection on a brief, lay-language summary of key TIF recommendations for transfusion in thalassemia; and (3) reaction to three methods for storing and sharing personal transfusion data: a smartphone app, a wallet card, or a patient or transfusion registry. These options were described on the handout with practice recommendations. To minimize the time burden on participants and staff, and to support participants' confidence in their anonymity, thus improving candor and reducing social desirability bias, the interview guide did not collect demographic information.

The same interdisciplinary team translated relevant TIF guidelines into the short, lay-language handout mentioned above (see Supplementary Material), which was shared with participants before asking questions about their experience of providers' following those practices. Accessibility to a wide patient audience was validated by a Flesch-Kincaid Grade level of 8.7. Medical accuracy was confirmed by clinical specialists in thalassemia and transfusion on the team. Patient advocates helped assure patient-centered language. The study was approved for human subject research through the Georgia State University Institutional Review Board and all participants gave informed consent.

### Recruitment

Inclusion criteria, all confirmed by self-report, included a thalassemia diagnosis, being at least 13 years of age, and having received at least one blood transfusion as a treatment for thalassemia. These criteria were chosen to capture a wide range of participants who could reflect on and communicate their experiences. The Cooley's Anemia Foundation, a nationwide thalassemia patient advocacy group, assisted in recruiting a convenience sample of participants meeting inclusion criteria. The Foundation shared the approved recruitment flier through its social media channels and email listserv. Interested individuals followed an embedded link to an online form where they provided availability and contact information. A researcher followed up to schedule the interview at a mutually convenient time. Consent was obtained at the time of the telephone interview. If the participant was a minor, a parent or guardian was required to be present for the interview and provide consent, while assent was obtained from the minor. Participants received a $25 gift card in consideration of their time.

### Data Collection

Data collection took place in June and July 2018. All interviews were conducted one-on-one in English, over the telephone from the work office of one of three researchers. The goals of the study were explained prior to the commencement of the interview, with consent obtained to continue. Interviews took ~30–40 min and were audio-recorded. Recorded interviews were transcribed verbatim and de-identified for review and analysis. Interview recordings and transcripts with personal identifiers were stored on a secured server in password protected files accessible only to IRB-approved researchers.

### Analysis

Data analysis was guided by the “Sort and Sift: Think and Shift” method (14, 15). This qualitative approach combines tenets of phenomenology, grounded theory, and narrative inquiry, all of which use an iterative process for data analysis that facilitates the merging of findings among the research team. The analysis followed five steps: (1) create a neutral domain name corresponding to each interview question; (2) create a summary template for use by the team; (3) assess the summary template; (4) establish consistency in template use across the team; and (5) transfer summaries into a matrix with rows for each respondent and columns for each domain to synthesize results (14).

Researchers created short domain names corresponding to each interview question. For example, the question, “What information have you been given about transfusions?” was represented by the domain name “transfusion information received.” Next, an interview summary template was drafted to include each domain name. Team members used this template to code an initial set of transcripts. The team met to determine whether the domain names were intuitive, accurate, and complete; to assess the ease of use of the template and length of time required; and to reach consensus on guidelines for consistent coding and template use–for example, the type and length of quotes to include on the form. Finally, the team coded the remaining transcripts and compiled the results into a single document for synthesis. Results were summarized for each domain, noting both common and unique responses to reflect the breadth of perspective and experience across the sample.

## Results

### Sample Characteristics

Thirty-two individuals participated in the study. Though weighted to the East and West coasts of the United States, the sample included participants from across the country: Alaska, Arizona, California, Florida, Kansas, Maryland, Massachusetts, Michigan, Missouri, Nevada, New Jersey, New York, North Carolina, Pennsylvania, Tennessee, and the District of Columbia. State of residence and spontaneous comments indicate that the sample included participants from both high- and low-population density areas. All participants were able to communicate effectively in English. One participant was a minor, who participated with a parent present; all other participants were adults.

### The Transfusion Experience

Respondents shared reflections on their experience before, during, and after receiving a blood transfusion. All participants were able to describe the frequency of their transfusions, the blood typing and crossmatching process prior to transfusion, and at least some potential transfusion complications, most notably iron overload. They described the process as lengthy, while also acknowledging the importance of transfusion for their well-being.

#### Frequency

Most participants stated that they receive transfusions every 2–4 weeks, although one participant described receiving transfusions when they feel like they need to or when they are “low on blood”. All participants managed the scheduling of their transfusions themselves, in consultation with their provider.

#### Blood Typing and Crossmatching

On average, participants described receiving blood typing and crossmatching 1–3 days before the transfusion. One participant explained, “I go into the hospital, and they already have the blood ready for me, because I was typed and crossed 2 days prior”. Another participant said they “go into the lab 2 days prior to my transfusion to get my blood drawn so they can crossmatch me, because I have antibodies in my blood and it's harder for them to find blood to match. So they need that extra day for me.” Another participant, who reported having received blood transfusions for 29 years, described a specific schedule: “I go on Tuesdays for...crossmatching, and then Fridays for the blood”.

#### Length

Most participants described long wait times during the transfusion: “They should get me a chair and it's about three and a half hours, I sit there, and I get the blood”, said one participant. Another said, “It's time consuming. I have to make sure... I have the day off, or I can...be able to be in the hospital all day.” A participant who reported receiving transfusions from the same hospital since the age of five described planning for activities to do during the transfusion. Their suggestion: “Bring like fun things to do, because you're... just gonna sit in a bed or a chair for the next 6 h, and it's a little boring.” Respondents mentioned doing work, watching TV, and “hanging out” as ways they pass the time during transfusion.

#### Complications

All participants acknowledged potential risks of receiving blood transfusions, most commonly iron overload. One explained, “Some of the bad things are iron overload. I already have a huge iron overload in my body, and so, you know, getting transfusions is just adding to it”. Some participants suggested ways to improve the transfusion process to address safety or convenience issues, though acknowledging that some of the ideas might not be feasible. Suggestions included improving tests for viruses in donor blood; transfusing with blood that has the iron removed; transfusing with blood that has extended blood cell life; receiving transfusions at home; and any methods that could shorten the transfusion process.

#### Importance

Most participants expressed that for them, transfusions are essential for staying alive. One stated, “You have to get blood. You don't have a choice, you know... If you don't get blood, eventually your blood count will go so low that, you know, you will die without the blood.” A few participants also described feeling better after individual transfusions, as in this comment: “Before transfusion I get really tired and barely can focus; and after I get transfusion, I'm more energized and I can focus better”.

#### Advice and Concerns

All participants shared advice on what a patient should know before receiving a transfusion. Most said that patients should be informed about the process, possible complications, and what the patient can do to avoid complications. A participant noted, “It's an important part that every patient knows what's happening, what pre-medications are you getting, what type of blood are they getting, just be willing to ask questions. And then, also, to know their own blood type and their own orders, so that if anything is different from what they were expecting, again, they can ask the question.” Other recurring sentiments were that a patient should “just kind of relax,” “be prepared to be there for a long time,” and again, “Don't be afraid to ask questions”.

Some respondents expressed satisfaction with their care and viewed their experiences positively. Others shared concerns about a lack of specialized medical providers and resources, particularly in some geographic areas. One participant said that this makes it even more important for the patient to be involved in their care by asking important questions. “I would be worried getting a transfusion at a small-town hospital. I guess those people, if they're doing that, they just really need to ask those questions that you asked me: Are they processing it this way and that way? And they need to know what diseases they're checking the blood for”. A few participants suggested a need for more providers who are knowledgeable about thalassemia. One said, “It's the subpar training of nurses or physicians. If you're in a smaller town, and I'm sure it's even worse in towns that are smaller than mine, just a lack of knowledge that people, you know, have had about this condition means you have to be your own health care advocate during blood transfusion”.

### Knowledge of Transfusion Recommendations

All participants were asked about five key TIF guidelines for reducing transfusion complications in transfusion-dependent thalassemia. The lay language versions of these are shown in Table 1 along with summarized results of patient knowledge of and experience with each. Most, but not all, participants were knowledgeable and aware of all five guidelines. Recommendations 3 and 5 were the least well recognized among the participants. Nearly one-third of participants (9 of 32) were unfamiliar with or unclear on the specifications “leukocytes removed,” “washed,” and “packed” red blood cell units. Some participants expressed a belief that recommendations 1 and 2 are standard procedure in all transfusion settings; however, the literature does not corroborate this (3, 16).

**Table 1:** Knowledge of transfusion guidelines.

**Guideline (lay version)**	**Summary of findings**	**Example quotes**
1)Wherever you go for a transfusion, doctors should have access to a record of your transfusion history. It should show up-to-date information on how often you get transfused, your blood type, and any antibodies or transfusion reactions you have developed.	Five of 32 study participants (16%) were unfamiliar with this recommendation. They agree that it should be practiced, with the understanding that it would be beneficial for both the patient and the provider. Many participants stated that the facility they go to for transfusions keeps a record of their history.	“No, I'm not [familiar with this recommendation]. I think that, of course, I think that should happen…”
		“I have to tell [my hemoglobin and ferritin levels] to my doctor. She doesn't see it on her own. Right now, I'm vocal, but, God forbid there is something too severe that happens …and if I'm not aware of all that, this is something they might not be seeing.”
2) Before each transfusion, they should run a full crossmatch and screen your blood for new antibodies.	Five of 32 study participants (16%) were unfamiliar with this recommendation and were unclear about what a full crossmatch is, or if it is practiced by their usual care facility.	“I know they do a cross match. I don't know when they do a cross match, is it a full cross match.”
		“I haven't [heard of this recommendation], but that would be good because I do have a lot of antibodies.”
3) You should be given packed red blood cells with leukocytes removed.	Nine of 32 participants (28%) were unfamiliar with this recommendation. They were unsure of what packed red blood cells are or whether that is what they receive; and/or they were unfamiliar with the term “leukocytes” and why they should be removed.	“Um I know I get washed, I don't know if I get packed. …But I don't know about the leukocytes.”
		“I'm not necessarily familiar enough to know in certainty that it, on, on the leukocyte piece- what the benefits are, are, would be to remove them or not remove them.”
4) If you have a fever or allergic reaction during a transfusion, then in the future when you get transfusions the doctor should give you acetaminophen (Tylenol) or diphenhydramine (Benadryl) first.	Only four of 32 study participants (13%) were unaware of this recommendation. One participant suggested that premedication should be given by discretion; that isolated reactions should not dictate perennial premedication.	“I would like, see how you go on your next transfusion. Go very slow in the beginning, see if you're making reactions. And then, from there just… Because, there's been times that I've had reactions, and isolations, and the majority of times I don't.”
5) If you have a severe allergic reaction, they should give you washed, packed red blood cell units any time you get transfusions again.	This recommendation was unfamiliar to nine of 32 (28%) participants, who did not know what “washed, packed, red blood cell units” are.	“That I didn't know. I didn't know there was washed red blood cells... If you had a reaction. I did not know that.” “I'm not familiar with that. I don't think I've experienced it....What are ‘washed RBCs?"

### Perspectives on Data-Tracking Approaches

Study participants were asked to consider three general methods of data tracking: a smartphone app, a wallet card, or a transfusion registry. Confidentiality and privacy were common concerns; however, they led respondents to different conclusions. Some expressed worry about their wallet or phone being lost or stolen or of the app being hacked. Some wondered about the security of a registry; one expressed the opinion that a registry might be more secure than a smart-phone app. Specific feedback on tracking methods is presented in Table 2.

**Table 2:** Perspectives on data-tracking methods.

**Method**	**Summary of findings**	**Example quotes**
Phone app	Participants generally expressed approval or interest in a phone app used to store personal blood and transfusion information. Some participants did, however, express concern about the extent of confidentiality and security of the app. Others saw value in having easy access to their information for themselves and their medical providers.	“So, a lot of times I will have to go to [a hospital]...but they don't necessarily know anything about me, and my record. And if it's late at night I can't get in touch with my doctor, you know, something like an app or something where you do have your medical history on hand is very important.”
		“Some people don't know what their blood type is or how many times they get transfusions. So, I think it would just be a good idea for them if they have the information on their phones.”
Wallet card	Participants expressed no uniform opinions about a wallet card having their blood and transfusion information. There was concern about losing a wallet card but overall, participants valued the role that a wallet card could play in case of emergency.	“In an emergency... that information would be available to an emergency responder. So, if someone is... in a car accident, and needed blood transfusions, I think the responding team or the ER knowing what type of blood that particular patient could or could not accept would be extremely important.”
Registry	Participants generally expressed approval or interest in an electronic registry used for storing medical information. Participants were told that, ideally, patient information would be accessible online from any location and they could choose to share their information with any health care provider. Participants supported having their information available so that they could receive proper treatment anywhere, especially when traveling. However, there was no consensus on whether the registry could be trusted to keep medical information confidential	“It's not that difficult to get into someone's cell phone… As opposed to a registry I think would have more security.” “As long as there's not, like, social security number or anything like that, I wouldn't worry about anybody getting into that.”

## Discussion

### Implications

The purpose of this study was to describe thalassemia patents': (1) knowledge and experience of transfusions and their complications; (2) knowledge of and experience in relation to their providers' adherence to recommended transfusion practices; and (3) preferences regarding possible methods to store and share personal transfusion data. Observed knowledge gaps, reported experiences, and the perspectives patients shared all suggest opportunities for intervention to improve transfusion safety.

### Transfusion Complications

Nearly all participants were aware of iron overload as a potential transfusion complication, and of chelation therapy to manage it. The clarity displayed by this majority about the importance of adherence to prescribed chelation therapy suggests that patients could be powerful spokespersons for delivering this message to peers. At the same time, the fact that a minority in the sample knew of this serious complication but not of its management indicates a need for increased education on chelation. Patients were familiar with concepts of mild transfusion reactions; but less mention was made regarding hemolytic transfusion reactions. This may suggest a need for more education among thalassemia patients about the potential for alloimmunization and the goals of antibody screens and crossmatching. Some concern was expressed about infection, a relatively low risk, suggesting that a realistic understanding of the degree of safety of the blood supply continues to be important for the thalassemia community, as it is for the public at-large.

#### Practice Guidelines

In rare diseases such as thalassemia, patient knowledge and self-advocacy can be important factors in receiving appropriate care. This makes translation of practice guidelines into lay language for affected populations a vital component of guideline dissemination and implementation for such conditions. Participants in the current study noted, for example, that those in small towns, where providers familiar with thalassemia may be far away, have increased burden for understanding their condition, knowing their medical history, and asking the right questions. Telementoring by peers could be a valuable resource for such individuals.

Most patients in the current study were aware of the five practice guidelines. Their misconceptions about the universal practice of the first two, as noted above, suggests that patients be encouraged to remain vigilant about them. Of all five recommendations, patients were most well-versed in the fourth one, regarding pre-medication to avoid allergic reactions. They were least well-versed in recommendations 3 and 5 regarding blood specifications. The need for education on these may reflect the technical terminology involved, as patients expressed unfamiliarity with “washed, packed” units as well as “leukocytes” and “leukocyte-reduced” units. The possible gaps in provider practices that are reflected in patients' reported experience are assessed more directly elsewhere, with provider-focused intervention recommendations (3, 16).

#### Data Tracking

The transfusion guidelines referenced in this study point to the need for providers to have accurate, up-to-date transfusion history for the patient on which to base clinical decisions. In the U.S., there is no health record universally accessible to providers across health systems. Providers seeing patients outside of patients' usual places of care must rely on patient report or real-time outreach to their prior provider. Data tracking approaches that improve on those two, flawed options could increase adherence to practice guidelines and reduce transfusion complications. A national antibody registry is the recommendation based on this study's findings. Privacy and security concerns were lowest for this option; it avoids the patient burden of keeping up with a wallet card or smartphone and installed app; and it would be fully accessible even in the event of a patient being unable to speak or operate their smartphone. Patients affirmed the urgency of closing the data gap, expressing doubt about the sufficiency of medical training in thalassemia; worry on behalf of patients in less-populated areas with even less access to knowledgeable providers; and acknowledgment of risk when receiving a transfusion away from the known provider. Developers of solutions should note the common concern for privacy among patients and account for this in both the design and the implementation of data sharing strategies.

### Limitations

Thalassemia is a rare disorder, with a relatively small patient population in the United States. While the present sample included individuals from multiple regions of the country and from both high- and low-population density areas, its size limits its representativeness of the overall study population. The absence of demographic data precludes characterization of the sample's representativeness across race, ethnicity, sex, and age. Because the thalassemia population in the U.S. includes both multi-generation Americans and more recent immigrants from a wide range of countries, the English proficiency of the current sample may introduce bias as well. Finally, recruitment through the Cooley's Anemia Foundation mailing list and inclusion by voluntary response may mean the sample skews toward being better informed on thalassemia and more engaged in personal care than the overall population of individuals with thalassemia receiving therapeutic blood transfusions. Despite these limitations, the range of responses and their convergent and divergent aspects offer findings that may be useful as a starting point for further studies or pilot interventions.

### Conclusions

The findings of this study can contribute to health education and other patient interventions, as well as future research initiatives, for reducing complications of therapeutic transfusions in individuals with thalassemia. While the study sample encompassed a generally well-informed group, gaps in knowledge and understanding emerged. These include the meaning and implications of terms such as “leukocyte-reduced” and “washed-packed” red blood cells; the recommended extent of antigen matching; and the balance between privacy concerns and life-threatening risk that attach to decisions about data storage to ensure accurate transfusion history for providers at the point of care. Increased education on these topics is recommended. Peer mentoring strategies that connect geographically isolated patients with others who have access to more robust thalassemia care and resources could help bridge an indicated gap. A national antibody registry is likely to find the greatest acceptance among patients, but care should be taken to address specific patient concerns in its design and implementation. Finally, a dissemination and implementation strategy for best practices and clinical guidelines—particularly in the case of a rare disease such as thalassemia—would do well to include parallel, patient-focused strategies.

## Data Availability Statement

The datasets presented in this article are not readily available because names and other identifying information will only be found on contact sheets, consent/assent forms, and gift card receipts, all of which will be accessible only to focus group facilitators and kept in locked offices at Augusta University and Georgia State University. Recordings will be stored on password-protected hard drive (R-drive) and GSU's password-protected OneDrive. The recordings on both drives will only be accessible to research staff with proper IRB approval. Research materials will be kept in locked offices or, if electronic, the Augusta University R-drive (hard drive) and GSU's password-protected OneDrive. Requests to access the datasets should be directed to Sashoy Patterson spatterson8@gsu.edu.

## Ethics Statement

The studies involving human participants were reviewed and approved by Georgia State University Institutional Review Board. Written informed consent to participate in this study was provided by the participants' legal guardian/next of kin.

## Author Contributions

AS, SP, and JB: study design. SP and VN: data collection. SP, VN, RS, and JB: analysis and writing. All authors contributed to the article and approved the submitted version.

## Funding

This work was made possible by cooperative agreement number NU58DD001138 from the Centers for Disease Control and Prevention. The content is the responsibility of the authors and does not necessarily reflect the official policies of the CDC or the Department of Health and Human Services.

## Conflict of Interest

The authors declare that the research was conducted in the absence of any commercial or financial relationships that could be construed as a potential conflict of interest.

## Publisher's Note

All claims expressed in this article are solely those of the authors and do not necessarily represent those of their affiliated organizations, or those of the publisher, the editors and the reviewers. Any product that may be evaluated in this article, or claim that may be made by its manufacturer, is not guaranteed or endorsed by the publisher.
