# Supplementary material for: Transfusion Complications in Thalassemia: Patient Knowledge and Perspectives
Source: Front Med (Lausanne). 2022 Mar 1;9:772886. doi: 10.3389/fmed.2022.772886 (PMC8923080; doi:10.3389/fmed.2022.772886)
Supplement: Supplementary file 1 [file Data_Sheet_1.PDF]

# Thalassemia Patient Interview/Focus Group Script

**Interviewer(s): ______________________________________________**

**Date/Time of interview/focus group: _____________________________**

**Number of participants: _____________________**

**Informed Consent:** For interview: Complete informed consent prior to interview. For group: As participants come to the focus group location informed consent will be completed prior to the start of the focus group.

**Introduction:** We would like to thank you for participating today. I want to begin by formally introducing myself and the other group leader. I am ________________ and this is _______________. You have all had a chance to read the consent documents and have signed them. Do you have any questions before we begin? For reasons of confidentiality, we will replace your name with a pseudonym in the transcripts. So, before we turn on the tape recorder, I would like to go around the room and have you introduce yourselves.

This focus group/interview should take between 60 and 90 minutes. Keep in mind there are no right or wrong answers. *Although you may hear opinions that you agree or disagree with the goal of the group is not to argue or change each other's minds. Rather, we hope that hearing many points of view will help you respond to the questions more completely.* Please be honest and answer questions openly. Your answers will be recorded, but none of the information will be linked to you. Your participation is completely voluntary, and you can stop any time, and no one will be upset with you. Do you have any questions before we begin?

**Question 1**: I would like to begin by asking *each of* you to briefly share a typical or usual transfusion experience. (This includes any preparation, travel)

*Probe: How often do you receive a transfusion? Who establishes that schedule? Do you go for cross-matching on the same day as your transfusion? If no, what do you do?*

**Question 2A:** What information have you been given about transfusions and who provided this information?

*Probes can ask about specific types of providers*

- *Hematologist, doctor, or transfusion clinic,*
- *Peer group friends, family members*
- *Support groups, health departments, patient organizations, blood banks*
- *Drug companies, pharmacists*
- *Websites, Facebook, chat rooms, social media*

**Question 2b:** Have you been given any information about iron overload and or chelation therapy?

*Follow up:* How would you rate the quality, timeliness, and importance of this information? Is there anything that would help you obtain more or better information?

**Question 2c:** What information about transfusion have your found on your own? Where have you looked? What were you looking for? Were you successful?

*Probe: Websites, Facebook, chat rooms, social media*

**Question 3:** What Are the good and/or bad things about receiving a blood transfusion?

*Probes: Do you have any concerns about blood safety? Is there anything that would make the process of transfusion better? How do you manage the transfusion experience?*

*What would make this process better? Is there anything else you would like to share with us about transfusions?*

**Question 4:** What do you think people should know before getting blood transfusions?

**Question 5:** The goal of this project is to develop ways and practices to make blood transfusions safer. I am going to share some of the ideas and practices that are being considered. **(Pass out handout with guidelines.)** I would like to hear your responses to these ideas and to then give you a chance to make suggestions of your own. What are your thoughts on the recommendations listed on the handout?

*Probe: Can anyone provide an example of how the recommendations were used during one of your medical visits?*

**Question 6: (Refer participants to handout to look at Phone app, wallet cards, and registry information.)** With the goal of helping to make transfusions safer, how would you feel about your personal blood and transfusion information being stored on a phone app, wallet card, or electronic registry so that you and doctors treating you could get access to this information anywhere?

**Question 6A:** If a registry were available,

- Would you want access to the registry?
- Would you trust this system to keep your personal medical information confidential?
- Would you allow your information to be stored?
- What would be the barriers to you participating in a transfusion registry?
- Do you have any other suggestions for tools to help with managing transfusions?

**Question 7:** Is there anything else you would like to share with us about transfusions?

(Do you have any additional ideas of what you and your medical providers can do to make transfusions safer?)

**Conclusion:** Conclude the Session by asking if there are any questions and thanking participants. Confirm email address for gift card.
