# Supplementary material for: Transfusion Complications in Thalassemia: Patient Knowledge and Perspectives
Source: Front Med (Lausanne). 2022 Mar 1;9:772886. doi: 10.3389/fmed.2022.772886 (PMC8923080; doi:10.3389/fmed.2022.772886)
Supplement: Supplementary file 2 [file Data_Sheet_2.PDF]

Reducing Complications in Transfusion-Dependent Thalassemia

Below are some recommendations from experts that doctors should follow when they care for people with transfusion-dependent thalassemia. Please read the recommendations and the things we are thinking of doing to help patients and doctors follow the recommendations. We would like to get your input on what we should do.

Antigen: A substance that your body sees as a threat. Your immune system creates antibodies to fight off the antigen.

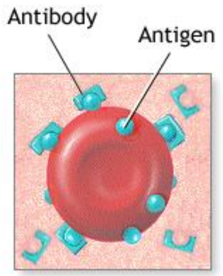

Red blood cell

Recommendations

(1) Wherever you go for a transfusion, doctors should have access to a record of your transfusion history. It should show up-to-date information on how often you get transfused, your blood type, and any antibodies or transfusion reactions you have developed.
(2) Before each transfusion, they should run a full cross-match and screen your blood for new antibodies.
(3) You should be given packed red blood cells with leukocytes removed.
(4) If you have a fever or allergic reaction during a transfusion, then in the future when you get transfusions the doctor should give you acetaminophen (Tylenol) or diphenhydramine (Benadryl) first.
(5) If you have a severe allergic reaction, they should give you washed, packed red blood cell units any time you get transfusions again.

Possible Activities

(1) **App:** This is an app for your phone to store your transfusion information. No one but you can see it unless you show it to them. You and your doctor enter the information in the app. It includes your blood type, antigens, past transfusions, and any reactions you have had to transfusions. It also has phone numbers for your doctor and the centers where you have had transfusions.
(2) **Card**: This is a card with your transfusion information for you to carry in your wallet or purse. No one else can see it unless you show it to them.
(3) **Registry**: This is a system for storing specific health information on a group of patients. It lets you look up your own information from anywhere using the internet. If you give them permission, it also lets a doctor look up your information so they can give you the right treatment.

Georgia Health Policy Center
GRU Georgia Regents University
Children's Healthcare of Atlanta
Georgia Southern University Jiann-Ping Hsu College of Public Health
every drop counts — Cooley's Anemia Foundation
The Sickle Cell Foundation of Georgia, Inc.
Emory Center for Transfusion and Cellular Therapies
Grady
DPH Georgia Department of Public Health
